# Nowhere to Play: Available Open and Green Space in Greater London Schools

**DOI:** 10.1007/s11524-021-00527-0

**Published:** 2021-03-19

**Authors:** Niloofar Shoari, Majid Ezzati, Yvonne G Doyle, Ingrid Wolfe, Michael Brauer, James Bennett, Daniela Fecht

**Affiliations:** 1 MRC Centre for Environment and Health, Department of Epidemiology and Biostatistics, School of Public Health, Imperial College London, London, UK; 2 Public Health England, London, UK; 3 Department of Women and Children’s Health, School of Life Course Sciences, Faculty of Life Sciences and Medicine, King’s College London, London, UK; 4 School of Population and Public Health, The University of British Columbia, Vancouver, Canada

**Keywords:** Schools, Green space, School grounds, London, Inequalities

## Abstract

Experiencing outdoor space, especially natural space, during childhood and adolescence has beneficial physical and mental health effects, including improved cognitive and motor skills and a lower risk of obesity. Since school-age children typically spend 35–40 hours per week at schools, we quantified their access to open (non-built-up) space and green space at schools in Greater London. We linked land use information from the UK Ordnance Survey with school characteristics from the Department for Education (DfE) for schools in Greater London. We estimated open space by isolating land and water features within school boundaries and, as a subset of open space, green space defined as open space covered by vegetation. We examined the relationship of both school open and green space with distance to Central London, whether the school was fee-paying, and the percentage of pupils eligible for free school meals (as a school-level indicator of socioeconomic status). Almost 400,000 pupils (30% of all pupils in London) attended schools with less than ten square metre per pupil of open space—the minimum recommended area by DfE—and 800,000 pupils attended schools with less than ten square metre per pupil of green space. Of the latter, 70% did not have any public parks in the immediate vicinity of their schools. School green space increased with distance from Central London. There was a weak association between the school-level socioeconomic indicator and the amount of open and green space. Fee-paying schools provided less open space compared to non-fee-paying schools in central parts of London, but the provision became comparable in suburban London. Many London schools do not provide enough open and green space. There is a need to ensure regular contact with green space through safeguarding school grounds from sales, financially supporting disadvantaged schools to increase their outdoor space and providing access to off-site facilities such as sharing outdoor space with other schools.

**Supplementary Information:**

The online version contains supplementary material available at 10.1007/s11524-021-00527-0.

## Introduction

The physical environment experienced during childhood and adolescence has profound impacts on health and wellbeing during the life course [1, 2]. Pupils typically spend considerable time in schools (35–45 hours per week); thereby, enhancing school physical environments can positively affect pupils’ health, wellbeing, and learning experiences [3–5]. In this respect, school grounds are valuable assets that play a key role in school-based health promotion and which may also provide benefits to surrounding neighborhoods and their populations.

There is growing evidence that outdoor open (non-built-up) space and particularly open space covered by vegetation (green space) has beneficial impacts on physical and mental health of children and adolescents. Outdoor space—even with its risks [6]—is essential for children’s healthy development through providing learning opportunities, the freedom to be active, and an environment for social interactions [7, 8]. Outdoor space that is natural, and green space specifically, has been associated with improved general health and wellbeing [9, 10], neurodevelopment and cognitive skills [11–13], social, emotional and behavioral development [14–16], academic performance [17, 18], mental health [19, 20], immune system [21, 22], motor development [23], physical activity [24–26], and decreased risk of being overweight [27, 28]. The presence of green space can also serve as a protective buffer against air and noise pollution in school grounds [29, 30].

Numerous studies investigated the provision of green space in terms of type, quality, and accessibility for adults [31–34] but fewer considered school-age children or the school environment specifically. Evidence on the beneficial effects of outdoor space brings schools to the forefront of health promoting interventions in a sustained, equitable, and efficient way [35]. Generally, studies on health outcomes with consideration of green space at schools are limited and have used different metrics to characterize green space, depending on the research question, data availability, age of children, and city-specific attributes. Some studies have used land cover data [36–38], normalized difference vegetation index (NDVI) [12, 39], tree canopy [40, 41], and physical inspection of school campus [18] to measure green space within school premises. Others have focused on the function of green space. Li and Sullivan [4], for example, showed that views of greenery from classroom reduced stress among high school pupils. Yet, no comprehensive study exists to assess the per-capita outdoor open and green space presence at schools, their spatial distribution, and social inequalities across a large metropolitan area like London. As cities increasingly become home to a majority of the world’s population [42], this knowledge gap limits the ability of cities to leverage schools as assets, supporting an entire generation’s health from early childhood. Poor access to outdoor space in childhood can deliver a generation with more health problems and greater need for treatment later in life.

In this paper, we integrated multiple city-level data sources to quantify outdoor open and green space within school premises in Greater London and its variations and inequalities by school location and school-level socioeconomic status.

## Methods

### Overview

We quantified school open and green space in 33 boroughs in Greater London, covering more than 1.3 million pupils. We integrated and analyzed publicly available land use data provided by the UK Ordnance Survey (OS), the most accurate and up-to-date source for geospatial data in Great Britain [43], and the Department for Education (DfE) within a geographic information system.

### Data Sources

#### Open and Green Space Data

Open and green space data was derived from OS MasterMap Topography Layer (version May 2019), a nationally maintained dataset that provides detailed geographic and attribute information on surface features, including buildings and structures, paths, roads, and natural environments. To identify open and green space within school premise, we used OS’s classification of topographic features. We used features under the theme labelled as “land”, which represent both human-made and natural features of surface cover. For example, playing fields, football pitches, areas of vegetation, basketball courts, and car parks are included under the “land” theme. OS then indicates whether these features are human-made or natural. Basketball courts, for example, fall under the category of human-made while football pitches with natural grass are classified as natural. The “water” theme includes all objects delimiting or containing water, including rivers, ponds, and swimming pools, that are further classified into human-made or natural (see Appendix 1 for detailed OS’s classification of the features included in this study).

#### Public Parks and Gardens

OS MasterMap Greenspace Layer (version October 2019) contains information on the location, extent, and function of green space that are accessible to the public such as public parks or gardens.

#### School Data

We used OS MasterMap Sites Layer (version October 2018), which maps the extent of educational establishments, to identify boundaries of school grounds in Greater London. Geographic school information was integrated with the DfE “get information about schools” register (downloaded in June 2019 from https://get-information-schools.service.gov.uk/), which provided information on establishment address, school type (e.g. fee-paying and non-fee-paying), number of enrolled pupils, and the percentage of pupils eligible for free school meals as a school-level indicator of socioeconomic status. We restricted our analysis to schools with pupils aged from 5 to 16 years and excluded schools that solely functioned as nursery, children centre, college, or university. More information on inclusion and exclusion criteria are presented in Appendix 2.

### Matching School Grounds with School Characteristics

To quantify per capita open and green space in London schools, we used information on the boundaries of school grounds and number of pupils currently enrolled. Since this information was provided in two data sources, data integration was necessary. We matched polygons from OS MasterMap Site Layer with data from DfE, which was geocoded via a point representing the centroid of school buildings. We developed a set of sequential algorithms to overcome the lack of a shared identifier in the two data sources. First, we employed a spatial point-in-polygon approach and ensured that matched point and polygon referred to the same school. Second, we matched by purpose-built identifiers that we generated by concatenating school name and their spatial attributes. Specifically, these identifiers included (i) exact school name and Lower Super Output Areas (LSOA), (ii) exact school name and London Borough, (iii) fuzzy school name and LSOA, and (iv) fuzzy school name and London Borough. Third, we spatially joined information from the two data sources such that each school polygon was given all the attributes of the point that was located within 70 m of the polygon edge if the school names matched. We selected this distance because we observed that it was less likely that point and polygon referred to the same school beyond 70 m. Details of the matching process are reported in Appendix 3.

### Statistical Analysis

We estimated open space by summing the area of features labelled as “land” and “water” that were delimited within each school polygon. We then quantified green space by isolating features under the class of “land” and “natural” and adding up their area within each school. In other words, green space was a subset of the natural features from the open space. We have focused on open and green space within school grounds and did not include land or assets outside school premises. This approach provides a detailed understanding of school space. By looking at randomly selected schools in Google Earth, we could distinguish whether schools’ sporting fields were covered with natural green space (and therefore included as green space) or they were asphalted (and included as open space). However, we were not able to differentiate between the type of green space, for example, whether it was grassland or covered with trees. Our analysis does not report blue space area because only a limited number of schools included water features. The amount of open and green space per pupil was calculated by dividing the corresponding absolute areas by the number of enrolled pupils. We examined how open and green space changes with distance from the official centre of London (Trafalgar Square). Finally, we investigated the amount of open and green space in relation to school type (fee-paying versus non-fee-paying), as well as with the percentage of eligible pupils for free school meals in non-fee-paying schools.

Schools might compensate for having no or limited open and green space by use of nearby public green space. Therefore, to capture green space in immediate vicinity of schools, we also identified the number of public parks and gardens within 100 m circular buffer around each school boundary [44]. We conducted the analyzes in the ArcMap v·10·5·1 (ESRI Ltd, Redlands, California) and R Statistical Software (Version 1·2·5001).

## Results

**Fig. 1:**
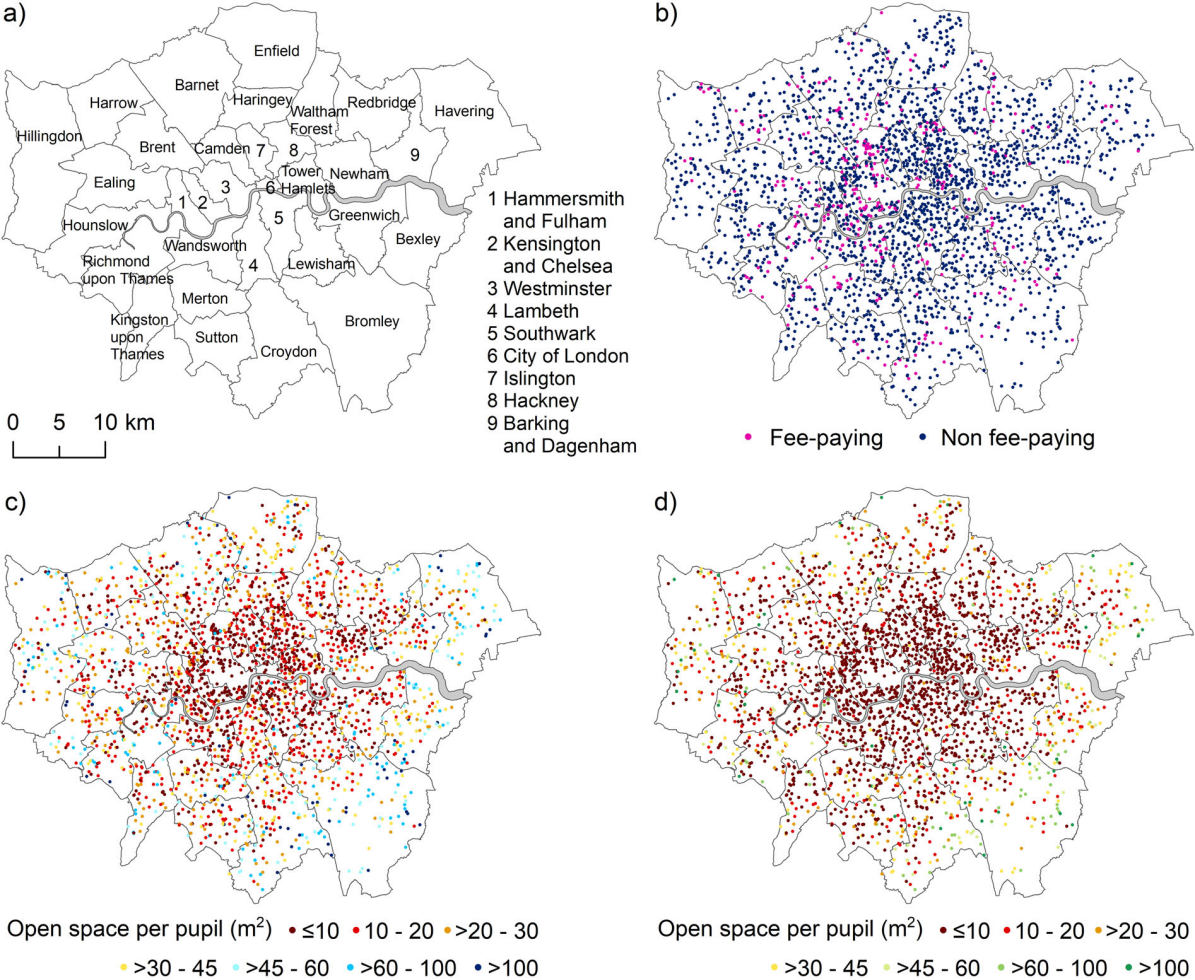
**a** London boroughs. **b** Location of fee-paying and non-fee-paying schools. **c** Open space per pupil for London schools. **d** Green space per pupil for London schools

**Fig. 2:**
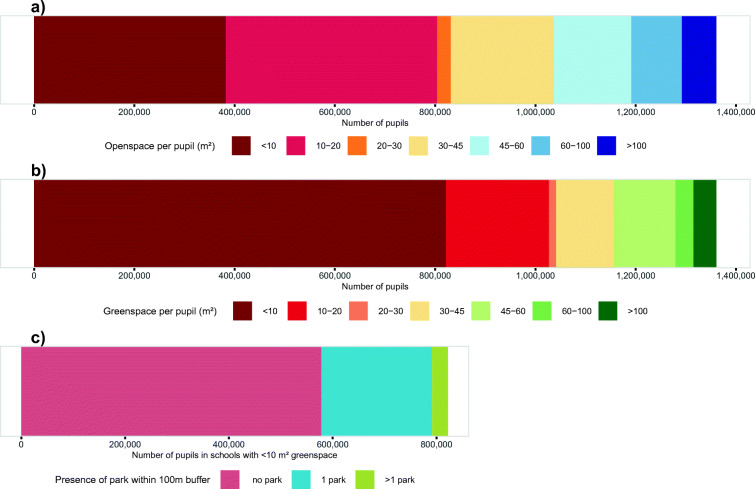
Number of pupils in different categories of **a** open space, **b** green space, and **c** public park availability when green space is < 10 m^2^

We estimated open and green space for 2,607 schools in 33 boroughs of Greater London, shown in Fig. 1a–b, stratified by school type (fee-paying versus non-fee-paying). Schools with limited open space were generally located in central parts of London (Fig. 1c). In the business-dense City of London, no school reached the recommended minimum of 10 m^2^/pupil of open space. The share of schools falling below this minimum was 79% in Westminster, 72% in Kensington and Chelsea, and 64% in Camden. Nearly 400,000 (~ 30%) of pupils in Greater London had less than 10 m^2^/pupil of open space available at their schools. With respect to green space, more than 70% of schools located in central boroughs of London (i.e. City of London, Newham, Haringey, Hammersmith and Fulham, Wandsworth, Lambeth, Southwark, Lewisham, Camden, Hackney, Tower Hamlets, Kensington and Chelsea, Islington, and Westminster) had less than 10 m^2^/pupil green space (Fig. 1d). More than 800,000 pupils (~ 60%) attended schools with less than 10 m^2^/pupil of green space, of which 70% (equivalent to 570,000 pupils) did not have any public park available in the immediate vicinity of their school to mitigate limited availability of green space (Fig. 2). More than 80% of schools with limited open and green space and no public park are located in the City of London and the borough of Kensington and Chelsea (see http://equitablehealthycities.org/focus-cities/london/london-schools-map/) for an interactive map of schools, their attributes, and nearby public parks.

**Fig. 3:**
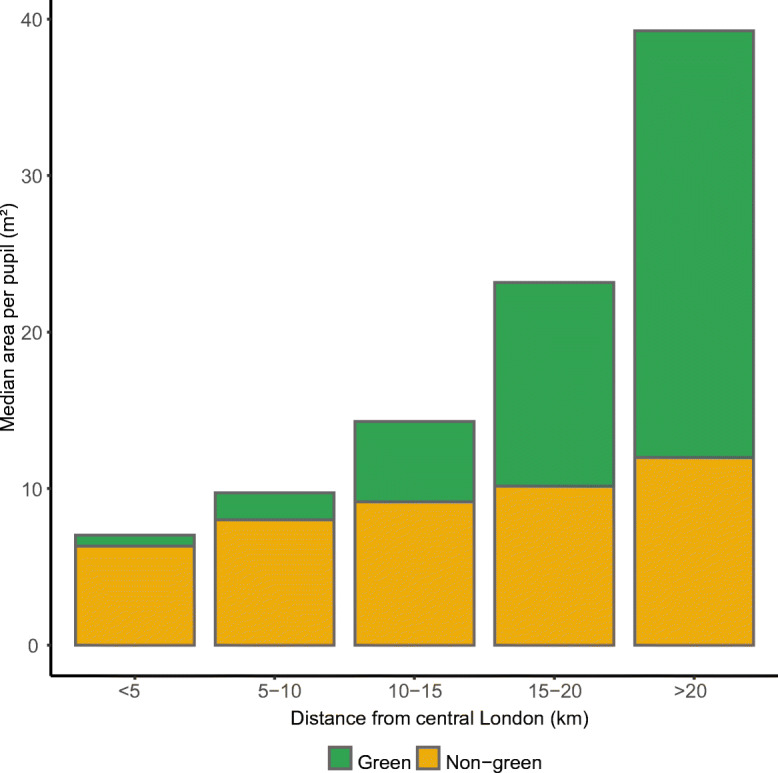
Median green and non-green space per pupil by distance from Central London. Open space is given by the sum of green and non-green space

The amount of open space per pupil increased significantly with distance from Central London. This increase was largely due to a rise in green space, with the non-green component of open space remaining almost the same across the entire Greater London (Fig. 3). Specifically, the median green area increased from less than 1 m^2^/pupil in central parts of London to 27 m^2^/pupil for schools located more than 20 km from Central London. Based on visual investigation of the function of outdoor space on Google Earth for a random selection of schools, those located in suburban London have generally a combination of green and non-green playing fields which in turn provide the opportunity for a diverse range of play activities [3]. Some examples of visual investigations are depicted in Appendix 4.

**Fig. 4:**
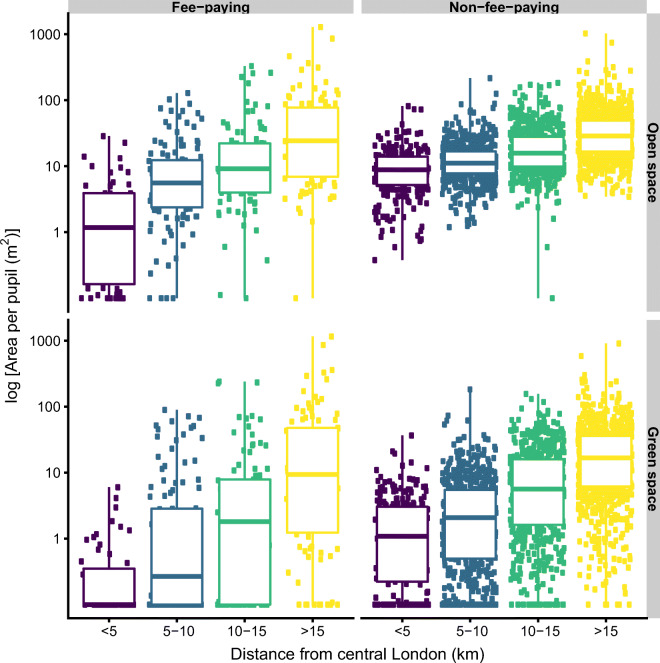
Open space and green space per pupil stratified by school type (fee-paying vs. non-fee-paying) and distance from Central London

Non-fee-paying schools generally provided more open and green space than fee-paying schools (Fig. 4). The median open space in non-fee-paying schools was 16 m^2^/pupil and only 7 m^2^/pupil in fee-paying schools. For green space, this difference became more marked with non-fee-paying schools providing on average 5 m^2^/pupil compared to 1 m^2^/pupil in fee-paying schools. Many fee-paying schools, especially those located in central parts of London, were small schools with no green space. It is only in suburban London (> 15 km from Central London) that green and open space area became comparable between the fee-paying and non-fee-paying schools.

**Fig. 5:**
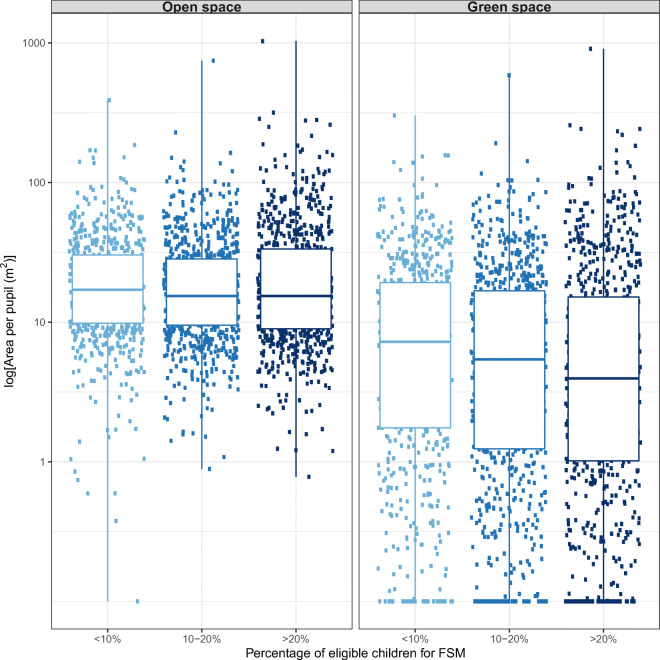
Open space and green space per pupil in non-fee-paying schools stratified by free school meal

Among non-fee-paying schools, the amount of open space did not vary substantially with the percentage of children eligible for free school meals; however, green space was slightly lower where a higher percentage of pupils were eligible for free school meals (Fig. 5).

## Discussion

More than 60% of children and adolescents in London do not have adequate open and green space at their school. Schools located in central parts of London generally do worse in terms of open and green space than those in suburban London, irrespective of the type and socioeconomic status of school. Further from Central London, schools are bigger and include green playing fields, while the non-green space remains largely the same across the city.

Many fee-paying schools are located in the areas of high land value, with no or small open space. However, fee-paying schools can mitigate the impact of limited space by offering a wide range of out-of-school activities [45]. Children from affluent families are more likely to participate in those activities and to have family-initiated activities, compared to their poorer peers on free school meals in non-fee-paying schools [46, 47]. Therefore, the variation in school open and green space is likely to aggravate the existing inequalities in access to, and utilisation of, quality urban green space [48, 49].

Given the significant health implications of the school environment, a public health perspective should underpin strategies that make schools healthier for all children. Specific actions that can motivate and enact actions to enhance better access to open and green space include the following:

- *Incorporating school-level outdoor open space*, *and especially green space*, *in school health indicators*. School health indicators allow taking an inventory of health promoting policies, acknowledging positive actions, identifying schools in need of improving health promotion, and helping schools to benchmark themselves against others. However, to our knowledge, none of the existing indicators (for example, the UK’s National Healthy School Status) directly accounts for school outdoor open and green space. Including information on the provision of open and green space and time available to children to spend outdoor in school health indicators will create a system of accountability for individual schools as well as the overall education system to provide health promoting environments.
- *Developing regulations to protect*, *and ideally increase*, *open and green space*. The current UK regulation set out in the School Premises Regulation (SPR, 2012) calls for “suitable outdoor space” for physical education by taking into account the age, number, and sex of pupils [50]. Shortage of open space in schools in London is partially because of extensive sell-off of land between 1979 and 1997. Nonetheless, it is still possible to dispose or change the use of school land by obtaining consent of the Secretary of State for Education. There should be vigilance that the intended change of land use retains a minimum area of outdoor space. School green space should not be sacrificed to create more classrooms, artificial playing fields, and indoor facilities.
- *Mobilising finance toward* “*worst-off*” *schools for equal opportunity of access to green space during school time*. Shortage in open space is the main constraint of schools to provide green space. Increasing the available space to the minimum recommended area of 10 m^2^/pupil, reported in Building Bulletin 103 “Area Guidelines for Mainstream Schools” guidelines, London would require an additional 1,500,000 m^2^ of open space, equivalent to roughly 200 football pitches. The lack of space is exacerbated by limited financial resources available to schools. Allocating government funds to disadvantaged schools will prevent sell-off of outdoor space. Providing financial incentives to greening initiatives that are prioritized for schools with low socio-economic status would promote an equitable use of outdoor open and green space for pupils. These initiatives include tree planting, installing roof gardens, and creating shrubs and urban school gardens. Creating schools with green space that can be shared with the wider community not only improves pupils’ health but also helps in making cities greener and increasing social cohesion. Finally, when school assets do not fulfil the minimum recommended open space, government needs to allocate resources for providing regular access to off-site facilities through off-site playing fields, parks, and gardens, using nearby schools’ open space, and organising trips to nature.

Experiencing natural environment during childhood and adolescence is essential for life-long health [2]. We provide a baseline for the current situation of open and green space in London’s schools. Our results should motivate a debate among stakeholders to identify and prioritize schools “at risk” and support the above activities for creating a healthier environment for children. This can be achieved via stronger links and collaboration among key players including urban planners and designers, developmental psychologists, education officials, and public health experts to ensure that adequate and age-appropriate open and green space in schools is at the forefront of urban planning both in and around schools.

### Strengths and Limitations

The strength of this study lies in integrating city-level data provided by OS and DfE to create a dataset that contains school boundaries and their detailed characteristics. To our knowledge, this is the first study that estimates school-level open and green space throughout a major city.

The limitations of this study arise from data availability. First, there were occasional discrepancies between the feature description in OS MasterMap Topography Layer and how it is used. Some schools, for example, might use a part of an outdoor space as parking area that is not accessible to pupils although the main label of this area is land. Second, we could not include rooftop green space because OS MasterMap Topography Layer does not capture this type of space. Third, we did not have information on the quality and usage of open and green space which may affect their relevance and students’ experience. Finally, we did not have information about out-of-school sport grounds such as rugby, football, and cricket pitches.

## Supplementary Information

ESM 1 (DOCX 2301 kb)
