# Supplementary material for: Nowhere to Play: Available Open and Green Space in Greater London Schools
Source: J Urban Health. 2021 Mar 19;98(3):375–84. doi: 10.1007/s11524-021-00527-0 (PMC8190412; doi:10.1007/s11524-021-00527-0)
Supplement: Supplementary file 1 — (DOCX 2301 kb) [file 11524_2021_527_MOESM1_ESM.docx]

# **Nowhere to play: available open and green space in Greater London schools**

Niloofar Shoari; Majid Ezzati; Yvonne G Doyle; Ingrid Wolfe; Michael Brauer; James Bennett; and Daniela Fecht

**Content**

| **Appendix A.** Diagram of open and green space feature classification of OS Mastermap Topography layer | **Page 1** |
| --- | --- |
| **Appendix B**. Inclusion and exclusion criteria for data sources | **Page 2** |
| **Appendix C.** Flowchart of matching data from Ordnance Survey and Department of Education to identify school boundaries and their characteristics | **Page 3** |
| **Appendix D**. Aerial images of schools and land use data in Central and suburban parts of London | **Page 5** |


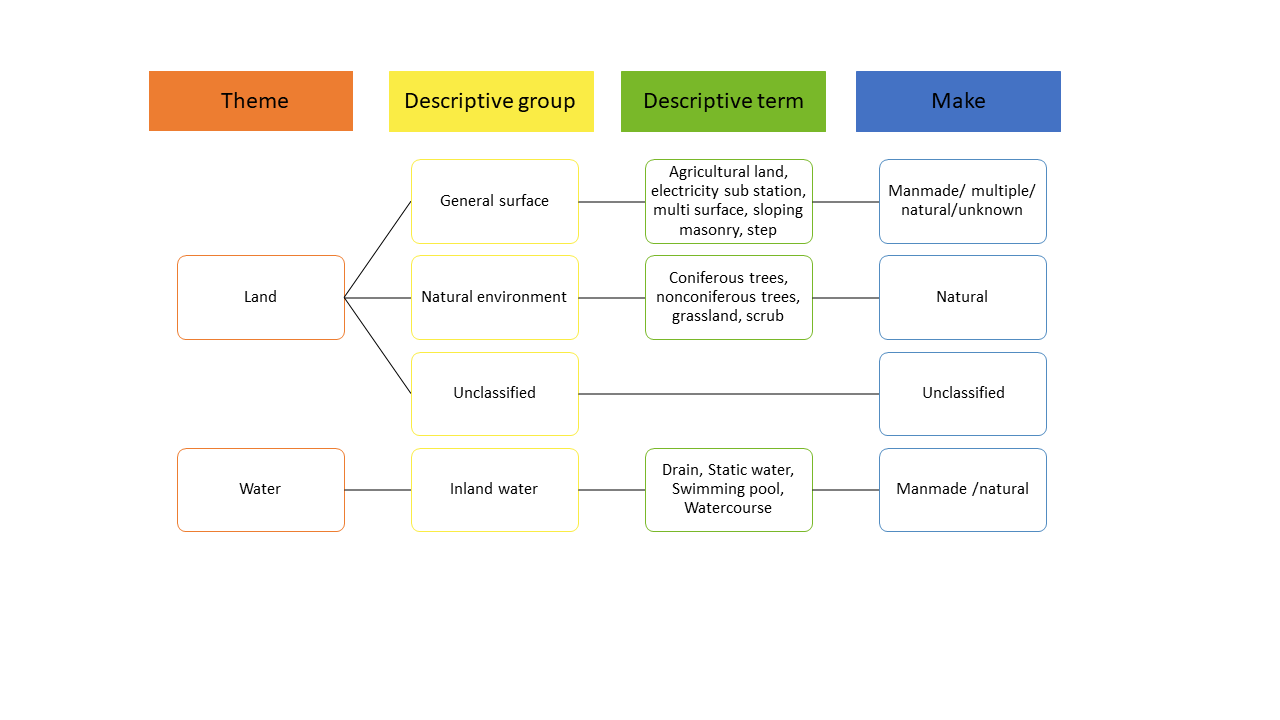


**Appendix A.** Diagram of open and green space feature classification of OS Mastermap Topography layer**.**

For ease of use, Ordnance Survey groups features of similar attributes into different themes. For this research, we used objects under land and water themes. The Land Theme represents “man-made and natural features that delimit and describe the surface cover, other than routes of communication and buildings” (Ordnance Survey, 2017). Water theme includes “features that contain, delimit, or relate to real-world objects containing water” (Ordnance Survey, 2017)

Ordnance Survey. 2017. OS MasterMap Topography Layer Product Guide v2. Available at <https://www.ordnancesurvey.co.uk/documents/os-mastermap-topography-layer-product-guide.pdf>


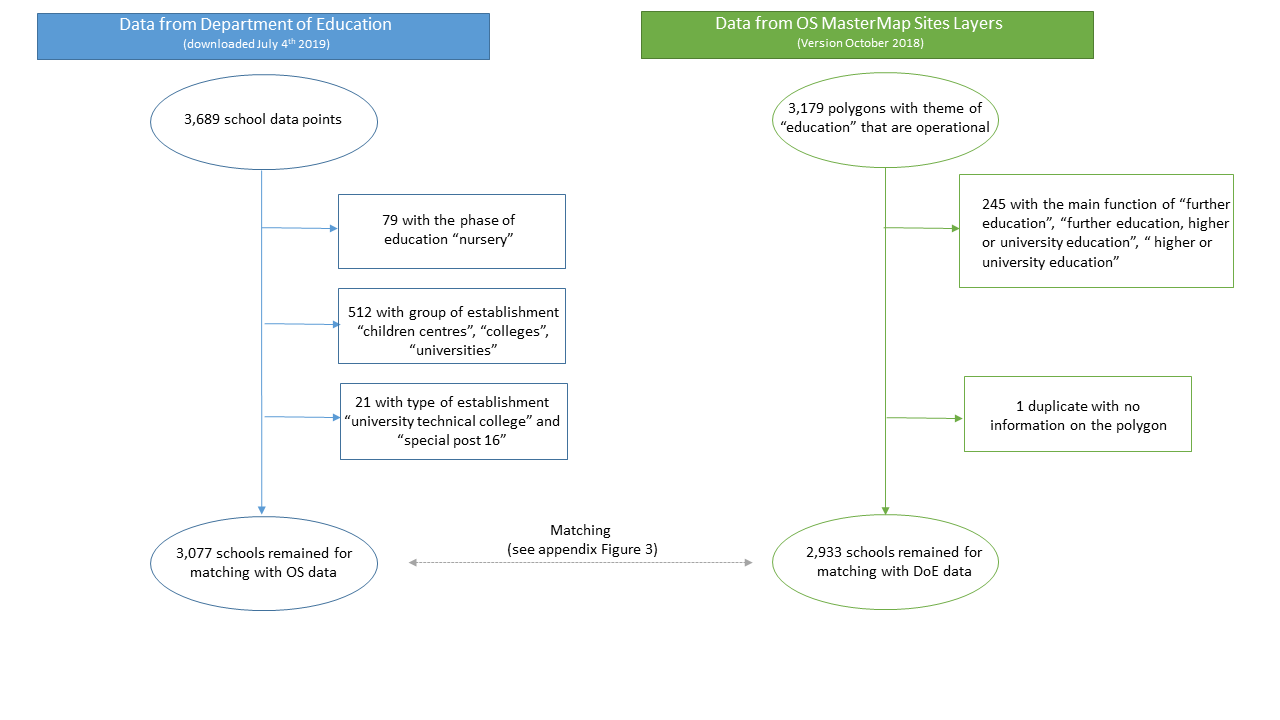


**Appendix B**. Inclusion and exclusion criteria for data sources


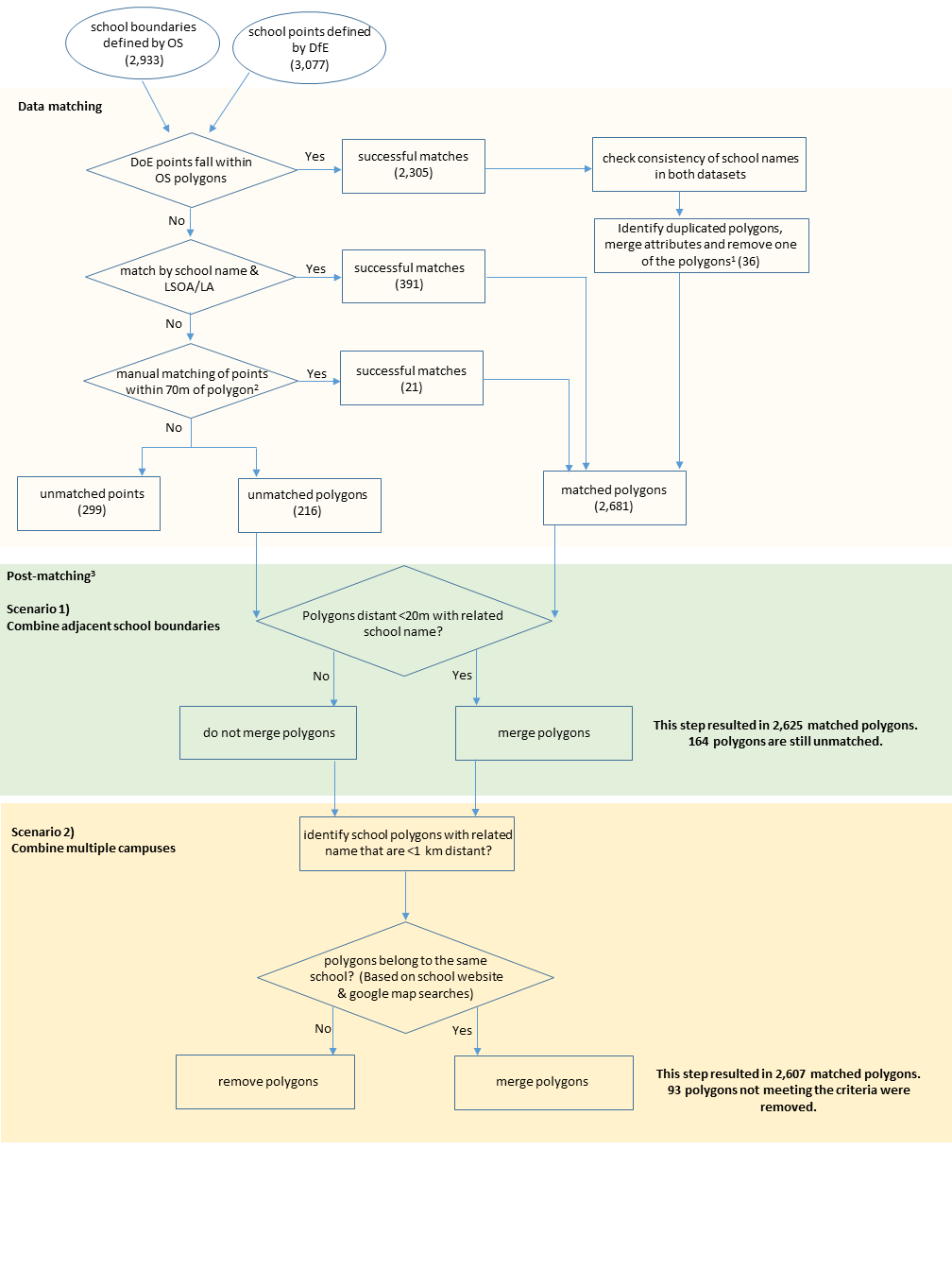


**Appendix C.** Flowchart of matching data from Ordnance Survey and Department of Education to identify school boundaries and their characteristics.

^1^ A typical situation where duplicated polygons occurred was when two same polygons represented different phases of the same school (for example, Trafalgar infant school and Trafalgar junior school). There were also a few cases where two same polygons belonged to different schools. In such situations, we removed one of the polygons but merged the attributes of them. Merging the attributes included combining school names, summing the number of enrolled pupils, and averaging the percentage of children eligible for free school meals.

^2^ At this step, we performed manual matching to deal with school name inconsistencies in datasets; these included contextual differences (e.g., German school vs. Deutsche Schule), truncated text (e.g., Coulsdon Church Of England Primary School vs. Coulsdon CofE Primary School), and prefix terms (e.g., Cumberland School vs. the Cumberland School).

^3^ Because the OS school polygons do not indicate legal extent but rather the extent of function, the following scenarios of challenge were evoked: Scenario 1) we identified cases where OS represented the subdivided parts of a school with two different polygons while DfE referred to the same school as a single entity. For example, Whitehall infant school and Whitehall junior school were represented by two polygons in the OS data whereas DfE referred to the same school with a single point labelled as Whitehall school. To deal with this issue, we assumed that it is quite likely that adjacent schools with related names share outdoor spaces. We devised an algorithm that identified schools that were either adjacent or within a 20m distance of each other and merged the boundaries if the names were related. Scenario 2) in schools with multiple campuses across London, only one polygon (i.e., campus) was matched. This happened because the OS data captures all campus polygons but the DfE data represents each school with a single point, which usually happens to be located in the main campus. Our algorithm identified potential schools with multiple campuses defined as polygons with related names. For these, we performed searches on the school websites and google map to verify the spatial location of campuses and merged campus polygons that were less than 1 km distant. We selected the distance of 1 km as it equates to approximately a 20-minute walk for an average child. We excluded the schools where the distance between different campuses was larger than 1 km because this is not a plausible distance to assume that pupils walk between campuses to use outdoor facilities.

1. **Example schools in suburban London**


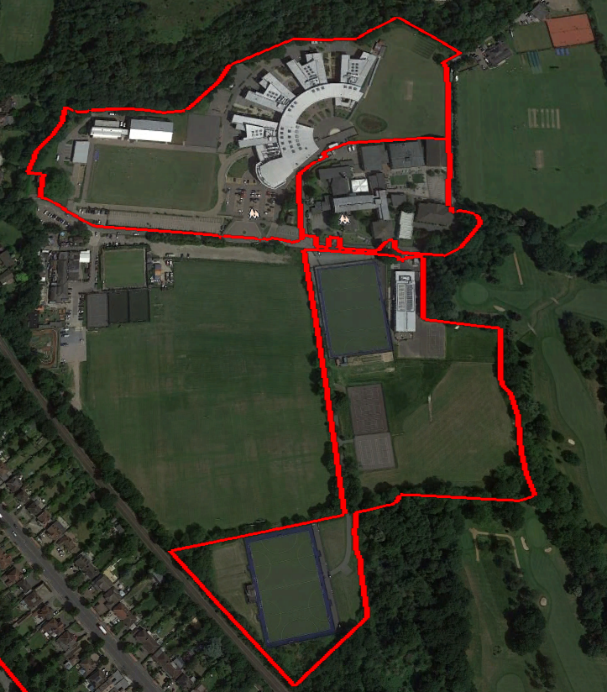

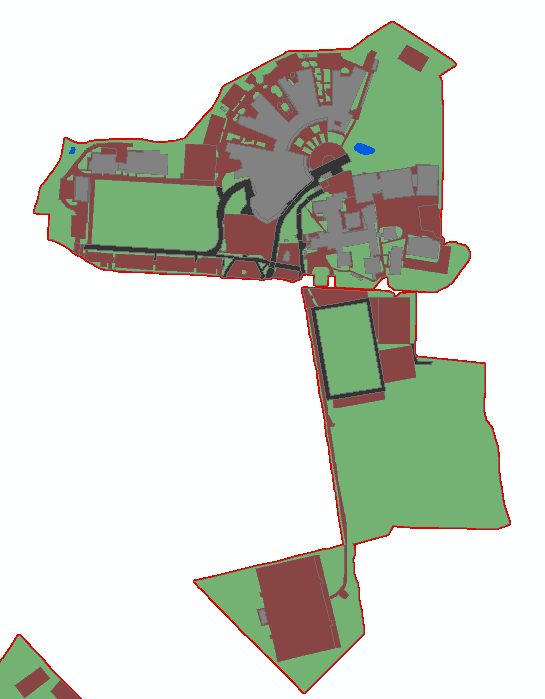


1. **Example schools in Central London**


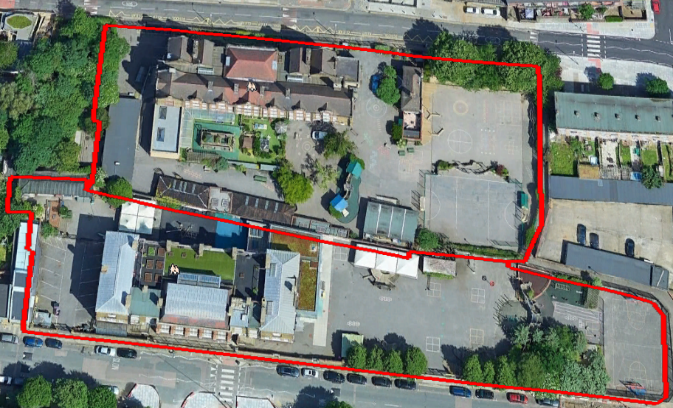

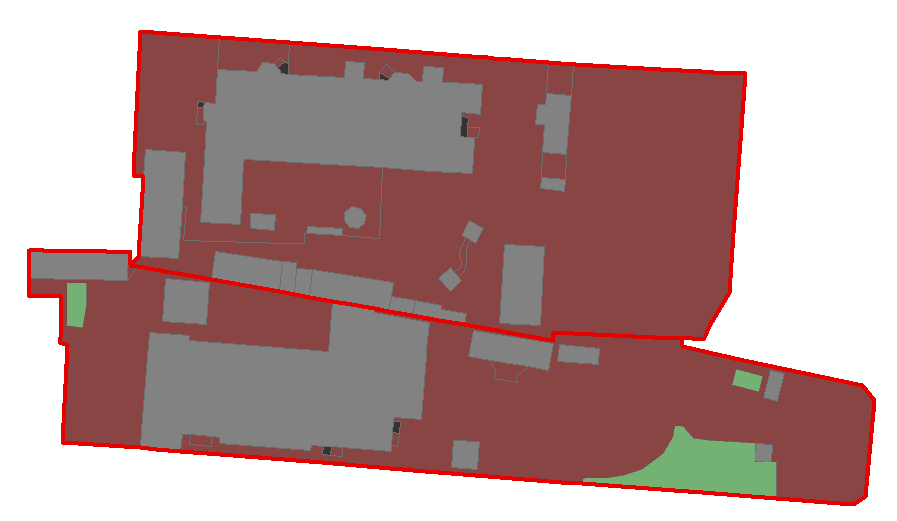


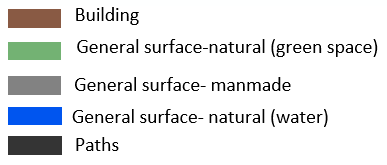


**Appendix D**. Aerial images of schools and land use data in Central and suburban parts of London
